# Effect of TiC on Microstructure and Properties of Wear-Resistant Mo_2_FeB_2_ Claddings

**DOI:** 10.3390/ma15134441

**Published:** 2022-06-23

**Authors:** Yiqun Sun, Junsheng Sun, Jun Jin, Hu Xu

**Affiliations:** 1 Laoshan Campus, Ocean University of China, Qingdao 266100, China; sunyiqun@ouc.edu.cn; 2 Key Laboratory for Liquid-Solid Structural Evolution and Processing of Materials, Ministry of Education, Shandong University, Jinan 250061, China; 200799013860@email.sdu.edu.cn (J.J.); xh0714@mail.sdu.edu.cn (H.X.)

**Keywords:** surfacing, Mo_2_FeB_2_, TiC, thermodynamic calculations, wear resistance

## Abstract

Alloy blocks with different TiC content were designed, and Mo_2_FeB_2_ cermets were prepared by carbon arc surfacing process. The interaction law of TiC content and the microstructure, phase, composition, hardness and wear resistance of the cladding were studied in detail by the combination of experiment and theoretical analysis. On the other hand, the phase transition process of the weldpool is theoretically analyzed by thermodynamic calculation method. XRD test results show that in addition to Mo_2_FeB_2_ synthesized in situ, the cladding also forms phases such as TiC, CrB, MoB and Fe-Cr. The number of Mo_2_FeB_2_ hard phases gradually increases when TiC content varies from 0% to 15%. The average microhardness of the cladding with 0%, 5%, 10%, and 15% TiC was 992 HV0.5, 1035 HV0.5, 1018 HV0.5 and 689 HV0.5, respectively, with 5% TiC being the largest. Moreover, the cladding with 5% TiC content has excellent wear resistance, which is 14.6 times that of the substrate.

## 1. Introduction

Transition metal borides are one of the hardest materials with high melting point, hardness and wear resistance. Therefore, borides are widely used in the field of wear resistance and have been intensively studied [1,2,3]. Metal-based ceramics such as Mo_2_NiB_2_, Mo_2_FeB_2_ and WCoB were initially prepared by Takagi using the reaction sintering technique [4]. Moreover, Mo_2_FeB_2_ has attracted widespread attention because of its low cost, excellent high-temperature hardness, strength, and good wear resistance and corrosion resistance [4,5,6,7,8]. Yu et al. used vacuum sintering technology to prepare Mo_2_FeB_2_ through the reaction 2Mo + 2Fe2B = Mo_2_FeB_2_ + 3Fe, which is a major breakthrough in the application of Mo_2_FeB_2_ [9]. To further improve the comprehensive properties, the alloying elements including Mo/B [10], Cr [11] and V [12] elements were added into Mo_2_FeB_2_-based cermet.

At present, Mo_2_FeB_2_ is mostly prepared by vacuum sintering technology [13], which is complicated in process flow, expensive in equipment and high in preparation cost, which greatly limits the application of Mo_2_FeB_2_-based cermet. Compared with the sintering process, the welding process has lower cost, simple operation, and does not require expensive equipment. The in situ synthesis of Mo_2_FeB_2_ by welding metallurgy technology has important engineering practical value. However, due to the high temperature and large temperature gradient in the welding pool, the Mo_2_FeB_2_ synthesized in situ in the weldpool is large in size and unevenly distributed, and there are many problems such as insufficient hardness of the claddings, poor wear resistance, high brittleness and easy cracking.

To control the morphology of Mo_2_FeB_2_, the author studied the influence of Rare Earth (RE) content on the microstructure, hardness and wear resistance of Mo_2_FeB_2_ claddings, discussed the mechanism of RE refining Mo_2_FeB_2_ hard phase, and achieved ideal results [14]. To further homogenize Mo_2_FeB_2_ in the surfacing metal more effectively, it is necessary to study other technologies. In the process of welding metallurgy, the melting point of TiC (titanium carbide) is 3140 °C. It can serve as a non-spontaneous nucleation core of Mo_2_FeB_2_ in liquid metal [15,16], which accelerates the nucleation and growth of Mo_2_FeB_2_ and makes up for the problems of short weld pool time and insufficient metallurgical reaction. In this paper, Mo_2_FeB_2_ alloy blocks with different TiC contents were prepared, and their claddings were prepared by carbon arc welding. The effect of TiC content on the microstructure, composition, phase transformation behavior, microhardness and wear properties of the claddings was deeply studied. The formation mechanism of the hard phase in the cladding was studied by thermodynamic calculation.

## 2. Materials and Methods

The raw materials used to prepare the alloy blocks were mainly FeB powder, Mo powder, carbonyl Fe powder, TiC powder and Cr powder. Table 1 shows the particle size and chemical composition of the alloy powder. The composition design (mass fraction) of the powders was 10% Cr-6% B-47.5% Mo-x% TiC +balance Fe, of which the additions of TiC was 0%, 5%, 10%, and 15%. By adjusting the powder ratio in Table 1 to meet the composition requirements of alloy powder block design, and the powder was mixed and ball milled by a QM-3SP2 planetary ball mill. The process parameters were 560 r/min and ball milling for 6 h. Sodium silicate, which accounted for 10% of total mass of the powders, was used as binder. After milling, sodium silicate was added to the powders and mixed well. The mixture was pressed into the alloy blocks with dimensions of 90 mm × 30 mm × 3 mm at a pressure of 50 MPa and the average density was 4.69 g/cm3. The blocks were dried at room temperature for 8 h and placed in a drying oven at 150 °C for 1 h. Obtain alloy block experimental materials. The average density of the alloy block was 4.69 g/cm^3^. The substrate used in the surfacing test in this study was Q235 steel, and its chemical composition (wt.%) is S and P not exceeding 0.045%, and 0.15% C, 0.5% Mn and 0.3% Si.

**Figure 1:**
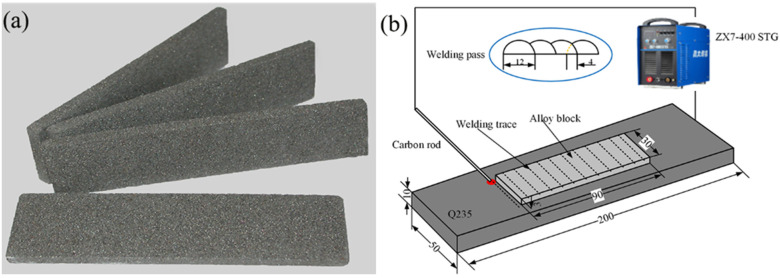
Alloy blocks and schematic diagram of the carbon arc surfacing (Unit: mm). (**a**) Alloy blocks, (**b**) Schematic diagram of the carbon arc surfacing.

Figure 1 shows alloy blocks and schematic diagram of the carbon arc surfacing. The size of the substrate Q235 for surfacing welding is 200 mm × 50 mm × 10 mm, and ZX7-400 STG inverter DC welding machine and Φ8 mm× 300 mm graphite rod were used. The graphite rod is clamped by the welding torch, and an arc is formed between the graphite rod and the substrate. The arc heat melts the alloy block and enters the weldpool, which reduces the burning loss of alloy elements. After the weldpool is solidified, the claddings deposited on the surface of the Q235 substrate was obtained. The process parameters of carbon arc surfacing are DC positive connection, rectangular swing, welding current 220~260 A, voltage 20~23 V, welding speed 100 mm/min. After the surfacing of the first layer is completed, after the sample is cooled to room temperature, use a wire brush to remove the slag on the surface of the cladding. In addition, using the same process as the first layer, the second layer is surfacing on the first layer, so that the cladding has enough thickness and reduces the influence of the dilution rate of the substrate on the cladding.

### 2.1. XRD Analysis and Microstructural Investigations

A typical cladding cross-section sample was cut from the surfacing specimen by wire cutting, ground with abrasive papers, and then polished with 1.5 μm diamond paste to obtain a metallographic sample. Then, it was etched with a volume ratio of 20 vol.% HF, 30 vol.% HCl and 50 vol.% HNO_3_ solution. The microstructure was studied with a scanning electron microscope (SEM) in backscattered electron (BSE) mode. The chemical composition analysis of the phases in the claddings was performed by energy dispersive spectroscopy (EDS) linked to SEM. The element distribution on the surface of the cladding layer was analyzed using a field emission electron probe (EPMA JXA-8530F PLUS). The X-ray diffractometer with Cu Kα radiation (λ = 0.154056 nm) was used to analyze the phase of the claddings, and the scan rate and scan step size were taken as 8°/min and 0.02°, respectively. The preparation of X-ray diffraction samples is the same as the above metallographic samples 

### 2.2. Hardness Measurement and Wear Tests

Along the depth direction of the cross-section of the cladding, the microhardness was tested by DHV-1000 micro-Vickers hardness tester. The distance between adjacent test points was 0.5 mm, the load was 0.5 Kg, and the dwell time was 10 s.

The size of the wear samples is 31 mm in the length, 5 mm in the width, and 7 mm in the thickness, i.e., 31 mm × 5 mm × 7 mm. Block on ring wear resistance tests were performed using an M-2000 testing machine under dry and rotating condition at room temperature. The material of the counterpart is carburized 20CrMnTi steel with a diameter of 40 mm, a thickness of 10 mm, and a Rockwell hardness of 60.2 HRC. The wear test conditions are that the rotation speed is 400 r/min, the load is 150 N, and the wear time is 60 min. For comparison, a wear test of the substrate under the same conditions was carried out at the same time. The mass loss value for each sample is the average of five experiments. When measuring the wear mass loss of the sample, the samples were first ultrasonically cleaned and then dried. Weigh the dried sample with an electronic balance and record. To determine the wear mechanism, the worn morphology of the claddings was analyzed by SEM.

## 3. Results and Discussion

### 3.1. XRD Analysis

**Figure 2:**
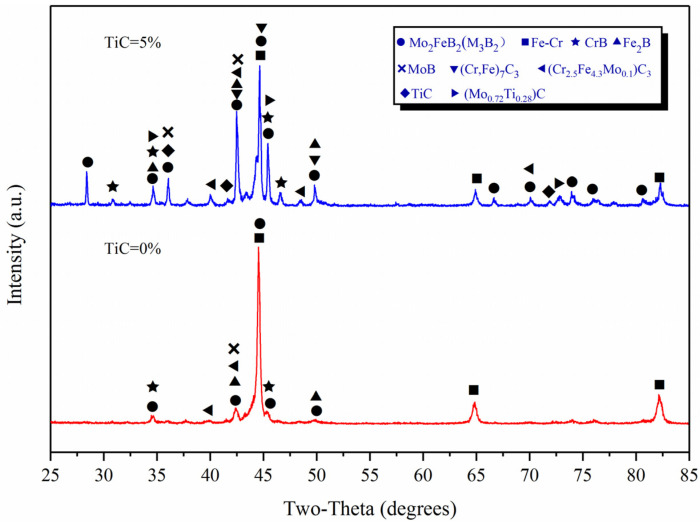
XRD analysis of claddings with 0% TiC and 5% TiC.

Figure 2 shows the XRD test results of the claddings with TiC content of 0% and 5%. It can be seen that the cladding layer is mainly composed of Mo_2_FeB_2_ (M_3_B_2_, M: Mo, Fe, Cr et al.) (JPCDS-18-0839), FeCr (JPCDS-05-0708), TiC (JPCDS-32-1383) and binary borides such as Fe_2_B (JPCDS-36-1332), MoB (JPCDS-06-0644) and CrB (JPCDS-26-0420). After TiC added, TiC and (Mo_0.72_Ti_0.28_)C (JPCDS-47-1078) appeared in the cladding. Composite carbides such as (Cr_2.5_Fe_4.3_Mo_0.1_)C_3_ (JPCDS-22-0211) and (Cr, Fe)_7_C_3_ (JPCDS-05-0720) were also found as strengthening phases. Please note that M_3_B_2_ is a complex ternary boride (Fe,Mo,Cr)_3_B_2_ formed by Mo_2_FeB_2_ and Cr at high temperature [17,18,19].

### 3.2. Microstructure and Composition

**Figure 3:**
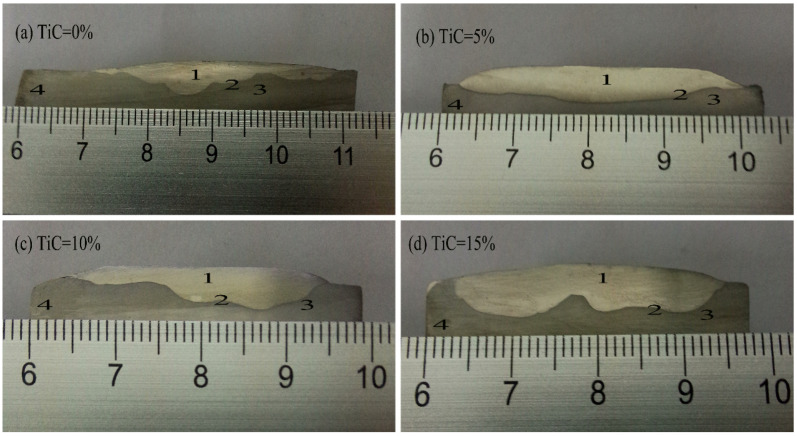
The macroscopic morphology of cross-section of with different TiC content 1: Cladding 2: Fusion line 3: Heat affect zone 4: Based metal (**a**) TiC = 0%, (**b**) TiC = 5%, (**c**) TiC = 10% and (**d**) TiC = 15%.

**Figure 4:**
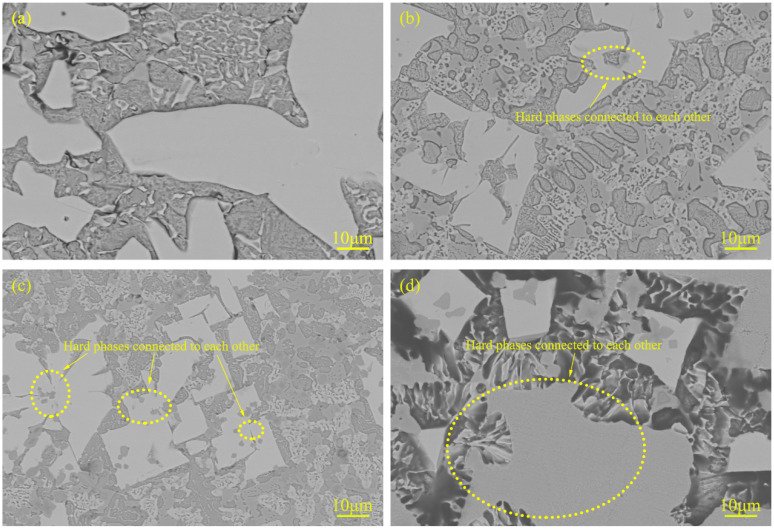
Microstructural morphology of claddings with different TiC contents (**a**) TiC = 0%, (**b**) TiC = 5%, (**c**) TiC = 10% and (**d**) TiC = 15%.

The cross-sectional macroscopic morphologies of surfacing metal with different TiC contents are shown in Figure 3. Typical microstructures of claddings with different TiC contents are shown in Figure 4. With the increase of TiC content, the number of white phases is increased. In addition, the white phases in cladding with TiC are finer compared with the cladding without TiC. This is because TiC has higher melting point and excellent high-temperature stability. The finely dispersed TiC particles play a role in pinning grain boundary migration during the growth of Mo_2_FeB_2_ [15]. At the same time, it can be seen from Figure 4b–d that some of the hard phases are connected to each other. This phenomenon can be explained according to the dissolution and diffusion mechanism [20], the white hard phases generated by in situ reaction nucleate, grow, and accumulate on the surface of TiC grains [16], which reduces nucleation resistance and nucleation work of the white phases, and eventually a large number of white Mo_2_FeB_2_ hard phases are formed and some of them are bridged together.

**Figure 5:**
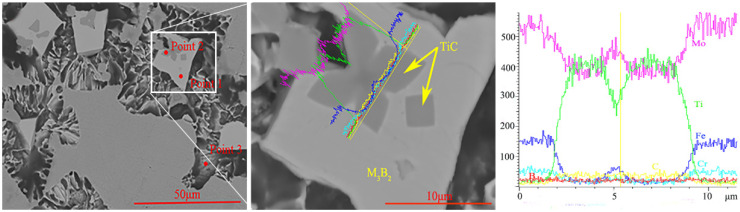
SEM analysis of claddings with 15% TiC contents and EDS line scan analysis.

Figure 5 shows the SEM microstructure of the cladding with 15% TiC content. Point 1 in Figure 5 is the hard phase, point 2 is the dark gray structure in the hard phase, and point 3 is the eutectic structure. EDS point scanning is performed on the above three points, and the test results are shown in Table 2. It should be noted that due to the low density of B and the insensitivity of EDS to B, the content of B was not accurate. The Ti content of point 1 and point 3 is less than 0.5%(wt.%), and the test results are also inaccurate due to the detection limit of EDS. Chromium replaces Mo and Fe sites in the Mo_2_FeB_2_ lattice to form the complex boride M_3_B_2_ (M: Mo, Fe, Cr) [18,21]. The atomic ratio Mo/Fe of the hard-phase Mo and Fe calculated from Table 2 is 1.7 (point 1), which is lower than 2, indicating that some Mo atoms in Mo_2_FeB_2_ are replaced by Cr atoms. Combined with the XRD results (Figure 2), it can be considered that the white hard phase is M_3_B_2_. Point composition analysis (Point 2) of the dark gray hard phase in M_3_B_2_ (white hard phase) in Figure 5 shows that C/Ti atomic ratio is about 1.5:1 not 1:1. The experimental conditions in this paper have the following two phenomena: first, because the cladding is made by carbon arc surfacing, some carbon elements will penetrate during process. Second, Due to its large affinity with oxygen, Ti has heavily oxidation loss during the cladding process, making it difficult to transition to the weld pool. Based on the above two reasons, C/Ti atomic ratio deviates from 1:1. According to the XRD results in Figure 2, it can be determined that the dark gray hard phase is TiC. During the formation of the liquid phase, TiC serves as the crystalline core and reduces nucleation resistance and nucleation work, which leads to ternary composite boride M_3_B_2_ grow around TiC (Figure 5). Moreover, the eutectic structure contains 27.45%C, 1.98%Cr, 60.2%Fe and 0.03%Ti (Point 3), illustrating that the eutectic structure contains (Cr,Fe)_7_C_3_ and TiC.

**Figure 6:**
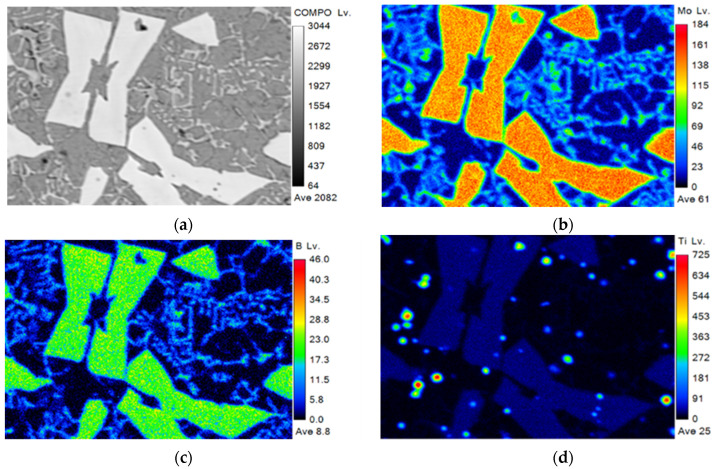
Element distribution images of cladding layer with 15% TiC added (**a**) BSE images (**b**) Mo (**c**) B (**d**) Ti.

Figure 6 shows the electron probe (FE-EPMA) test results of element distribution in the cladding layer with 15% TiC powder added. The results in Figure 6 show that Mo and B are the main constituent elements of the hard phase, which are mainly present in the hard phase. The Ti element is uniformly distributed in the hard phase, and aggregated into dots in the matrix and eutectic structure. This is consistent with the inference that TiC acts as the crystalline core, the hard-phase Mo_2_FeB_2_ grows around TiC, and the TiC generated around Mo_2_FeB_2_ inhibits the growth of the hard-phase.

### 3.3. Thermodynamic Analysis

TiC was added to the Mo-Fe-B alloy system through the metallurgical reaction of the weld pool, which contained phases such as TiC, MoB, CrB, and Fe_2_B in addition to the in situ synthesized Mo_2_FeB_2_ (Figure 2). Therefore, the main chemical reactions in the weldpool are shown in Table 3. The heat of reaction and Gibbs free energy [22] for each chemical reaction at different temperatures are calculated by Equations (1) and (2).

For the reaction heat at different temperatures and the Gibbs free energy [22], the results can be calculated by Equations (1) and (2).

(1) $$\Delta H_{T}=\Delta H_{298}+\overset{T}{\underset{298}{\int }}\Delta C_{P}dT$$

(2) $$\Delta S_{T}=\Delta S_{298}+\int_{298}^{T}\frac{\Delta C_{p}}{T}dT$$

(3) $$\Delta C_{p}=\sum {\left(C_{P}\right)}_{product}-\sum {\left(C_{P}\right)}_{reactant}$$

(4) $$C_{P}=a+b\times 10^{-3}T+c\times 10^{5}T^{-2}+d\times 10^{-6}T^{2}$$

where Δ*H*_298_ (J/mol) and Δ*S*_298_ (J/mol) are the standard enthalpy difference and standard entropy difference between the product and the reactant, respectively. *C_P_* (J/k·mol) and Δ*C_P_*(J/k·mol) are the standard constant molar heat capacity and the change in standard constant pressure molar heat capacity, respectively. *a*, *b*, *c*, *d* are the temperature coefficients of heat capacity of the substance.

(5) $$\;\Delta G_{T}=\Delta H_{T}-T\Delta S_{T}$$

(6) $$\Delta G_{T}=\Delta H_{298}+\int_{298}^{T}\Delta C_{P}dT-T(\Delta S_{298}+\int_{298}^{T}\frac{\Delta C_{P}}{T}dT)$$

where Δ*G_T_* (kJ/mol) represents the free energy change in products and the reactants at the reaction temperature *T*.

According to thermodynamic theory [23], the condition for the reaction to proceed spontaneously is Δ*G_T_* < 0.

**Figure 7:**
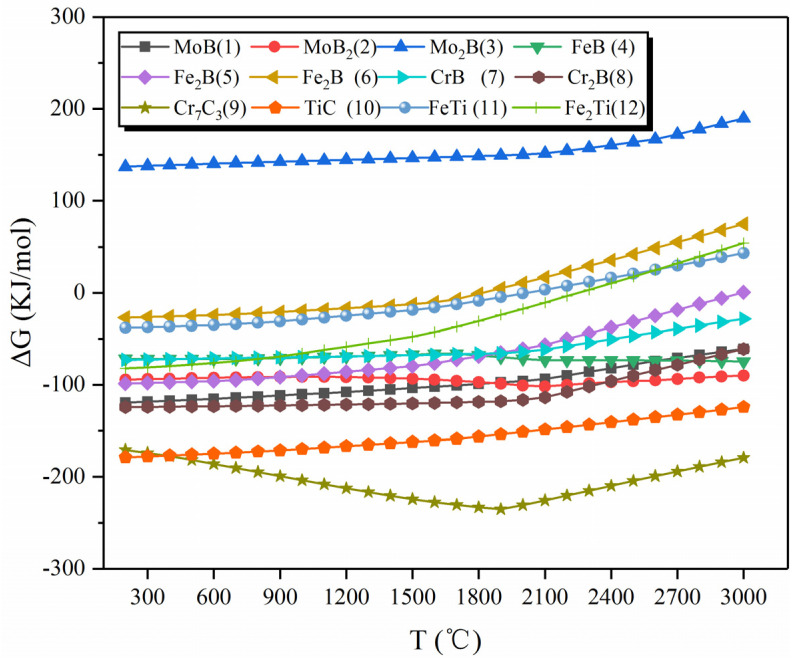
Effect of temperature on Δ*G* of each reaction.

According to the principle of minimum free energy and entropy increase, the Gibbs free energy change Δ*G_T_* of the reactions No. 1 to 12 in Table 3 is calculated by Equations (1), (2) and (5). To make the calculation results more accurate and convenient, this paper uses HSC chemistry software to calculate [24]. Figure 7 shows the calculation results. When temperature of weld pool is above 2400 °C, TiC, Fe_2_B and MoB are preferentially formed. When temperature drops from 2400 °C to 1800 °C, MoB and Fe_2_B continue to form and increase. Moreover, when temperature decreases from 1800 °C to 900 °C, the formation of CrB_2_ and MoB continues to increase. However, it should be noted that Mo_2_FeB_2_ phase was not directly formed from liquid phases. Ide et al. [25] reported that Mo_2_FeB_2_ was formed initially by the reaction 2Mo + 2FeB = Mo_2_FeB_2_ + Fe (No. 14 in Table 3) and later by the reaction of 2Mo + 2Fe_2_B = Mo_2_FeB_2_ + 3Fe (No. 15 in Table 3). Therefore, the amount of Mo_2_FeB_2_ produced by the reaction increases with the increase of FeB and Fe_2_B.

### 3.4. Microstructure Evolution

**Figure 8:**
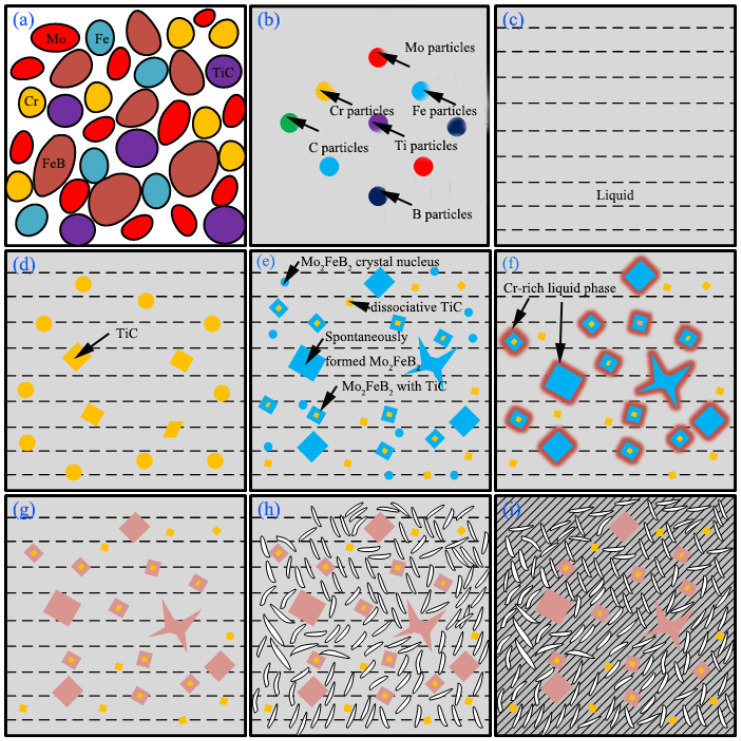
Schematic diagram of the microstructure evolution during the cladding process.(**a**) alloy powder (**b**) forming weldpool (**c**) weldpool (**d**) Nucleation and growth of TiC (**e**) Mo_2_FeB_2_ formation (**f**) Cr-rich around Mo_2_FeB_2_ (**g**) M_3_B_2_(M:Mo,Fe,Cr) formation (**h**) binary borides formed (**i**) eutectic transformation.

Figure 8 is a schematic diagram of the microstructure evolution during the cladding process. Figure 8a–c relates to the heating process, and Figure 8d–i correspond to the cooling process. Mixing well of powders at the first stage, as shown in Figure 8a. Figure 8b corresponds to the process of forming weld pool. Under the continuous action of the carbon arc heat source, the compounds in the alloy block begin to decompose. In addition, gradually change from solid to liquid, as shown in Figure 8c. In solidification stage, TiC began to nucleate and grow out during the cooling process of weld pool, consuming a large amount of Ti and C atoms in weld pool, as shown in Figure 8d. Subsequently, as temperature continues to decrease, Fe_2_B and MoB are precipitated attached TiC and react with Mo and Fe to form Mo_2_FeB_2_. Mo_2_FeB_2_ uses TiC as the crystalline core and grows in small massive characteristics. It should be noted that not all Mo_2_FeB_2_ hard phases are formed attached TiC as the crystalline core, and those Mo_2_FeB_2_ that TiC cannot serve as the crystalline core still undergo the nucleation-growth process. According to previous thermodynamic analysis, Mo atoms in the liquid phase react with FeB and Fe_2_B [No. 14 and 15 in Table 3] to form Mo_2_FeB_2_, the blue massive and butterfly shaped hard-phase Mo_2_FeB_2_, as shown in Figure 8e. The formation of Mo_2_FeB_2_ enriches Cr around the grains, forming a Cr-rich liquid phase region, as shown in Figure 8f. With the help of the high temperature of the weld pool and the concentration gradient of Cr, Cr in the Cr-rich liquid phase region diffuses into Mo_2_FeB_2_ [26], and replacing Mo and Fe atoms in the Mo_2_FeB_2_ lattice [18,21], as shown in Figure 8g. As the temperature continues to decrease, other binary borides formed (CrB), and B is basically involved in the formation of borides rather than solid solution in the matrix [27], as shown in Figure 8h. When the temperature of the weld pool dropped to the eutectic temperature, the remaining liquid phase would be converted to Fe-Cr and (Cr,Fe)_7_C_3_, as shown in Figure 8i.

### 3.5. Wear Characteristics

**Figure 9:**
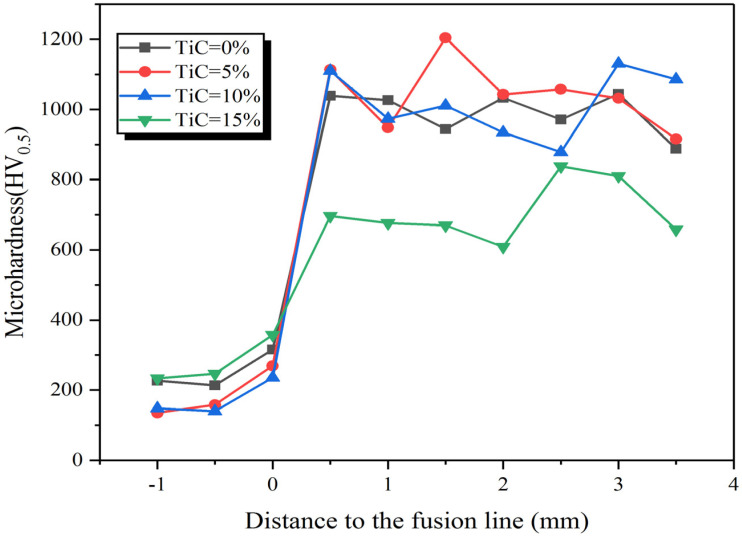
Microhardness of claddings with different TiC contents.

The average microhardness distribution of substrate and claddings are given in Figure 9. The average microhardness of the claddings with 0%, 5%, 10%, 15% TiC are about 992 ± 46 HV0.5, 1035 ± 37 HV0.5, 1018 ± 71 HV0.5 and 689 ± 101 HV0.5, respectively, which is 3.8~5.8 times that of the substrate (180 ± 32 HV0.5).

**Figure 10:**
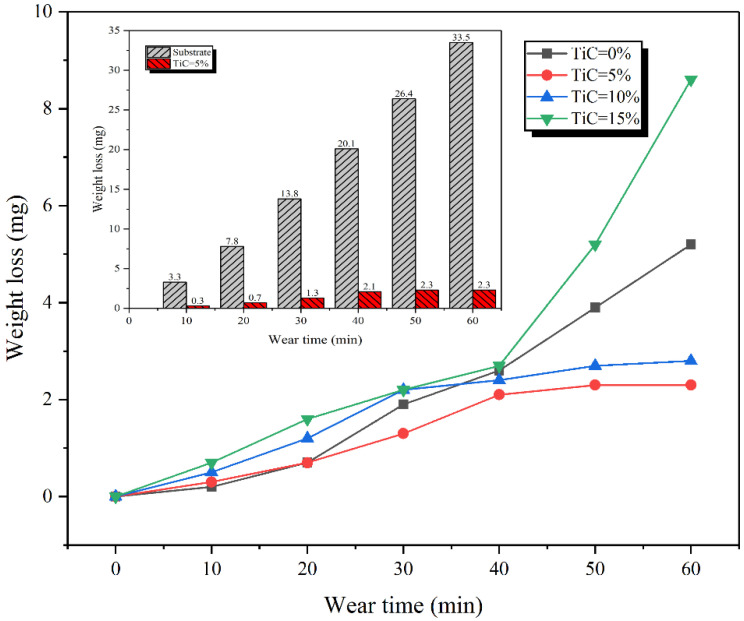
The relationship curve of wear loss weight with wear time of claddings with different TiC contents and substrate.

Figure 10 shows the relationship curve of wear loss weight with wear time of claddings with different TiC contents and substrate. The wear loss of cladding and substrate increased with the increase of wear time, but the increasing trend of the weight loss of the substrate was significantly greater than that of the cladding. Therefore, the wear resistance of the cladding is better. In particular, the cladding with 5% TiC content has excellent wear resistance (2.3 mg), which is 14.6 times that of the substrate (33.5 mg). The wear resistance of the claddings with TiC content of 5% and 10% is better. This is because the Mo_2_FeB_2_ in the claddings is large in number, small in size, and relatively uniformly distributed in the matrix [Figure 4b,c]. For the cladding with 15% TiC content, the wear weight loss begins to increase after 40 min. This is because bonding force between TiC particles and Mo_2_FeB_2_ is weaker and weaker as the wear test [15], and the bonding force of hard phase particles protruding from the surface was less than the shear stress on the wear surface. As a result, some of the hard phases are exfoliated from the surface, forming new abrasives that exacerbate the wear process.

**Figure 11:**
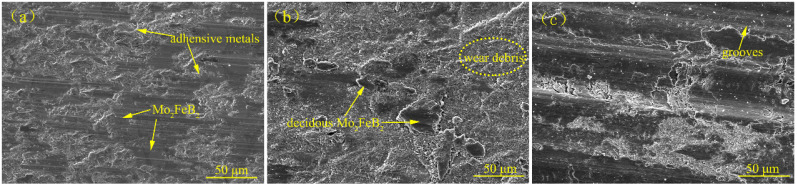
Wear morphology of claddings with different TiC contents and substrate. (**a**) 5% TiC, (**b**) 15% TiC and (**c**) Substrate.

Figure 11 shows the surface wear morphologies of the claddings with 5%, 10% TiC and substrate for 60 min. There is some adhering metal near the hard phase [Figure 11a], which indicates that slight abrasive wear occurs on the cladding surface. Adding appropriate TiC can fully use TiC as an inhibitor and a strengthening enhancer of Mo_2_FeB_2_ grain growth. Therefore, the claddings with 5% TiC are difficult to plough during dry sliding wear due to their high hardness. On the other hand, the Fe matrix prevents the hard-phase Mo_2_FeB_2_ from falling off and becomes newly abrasive by transferring the load and connecting the Mo_2_FeB_2_, which improves the wear resistance of the claddings. Mo_2_FeB_2_ bears most of the load by virtue of its high hardness, and the Fe matrix bears most of the plastic deformation. However, when TiC content reaches 15% [Figure 11b], wear debris accumulate around Mo_2_FeB_2_ phases and some deciduous Mo_2_FeB_2_ phases were observed. This is because with the increase of TiC content, the number and size of Mo_2_FeB_2_ phase increase, and the Fe matrix connecting Mo_2_FeB_2_ decreases relatively, which reduces the bonding strength of Mo_2_FeB_2_ phase and matrix metal, and the Mo_2_FeB_2_ phase falls off and becomes new abrasive during the wear process. The new abrasive that sheds during wear increases wear, which corresponds to the wear results of Figure 10. In addition, the hardness of the substrate is low because there is no Mo_2_FeB_2_ phase as a wear-resistant skeleton. The hard asperities on the surface of carburized 20CrMnTi can easily penetrate the contact surface of the substrate. When the substrate and 20CrMnTi move relative to each other, the wear surface of the substrate is prone to appear deep ploughing grooves [Figure 11c], the presence of deep ploughing grooves on the wear surface means severe abrasive wear.

## 4. Conclusions

1. TiC content had important effects on the microstructure of cladding. The number of Mo_2_FeB_2_ increased with the increase of TiC content. Moreover, hard-phase Mo_2_FeB_2_ in cladding with TiC are finer compared with the cladding without TiC.
2. TiC can serve as the non-spontaneous nucleation core of Mo_2_FeB_2_ in weld pool, which accelerated the nucleation and growth of Mo_2_FeB_2_ and made up for the problems of short weld pool time and insufficient metallurgical reaction.
3. The addition of TiC to the alloy blocks significantly improves the wear resistance of the claddings. When the TiC content is 5%, the cladding has excellent wear resistance, which is 14.6 times that of the substrate.
4. The results of this paper provide an effective technical solution for the in situ synthesis of Mo_2_FeB_2_-based cermets by welding metallurgy. Based on studying the influence of Rare Earth and TiC on the size, quantity, distribution and properties of Mo_2_FeB_2_, the effect of compound addition of Rare Earth and TiC needs to be further studied.

## Figures and Tables

**Table 1:** The particle size and chemical composition of the alloy powders.

Powder	Particle Size (μm)	Chemical Composition (wt.%)
Molybdenum (Mo)	75–120	Fe < 0.002,O < 0.1,Si < 0.001
Iron (Fe)	75–100	C < 0.1, N < 0.1, O < 0.2
Chromium (Cr)	90–150	O < 0.2, Fe < 0.18, N < 0.045
Ferroboron (FeB)	53–75	C < 0.27, Si < 0.71
Titanium carbide (TiC)	2–4	O < 0.06, S < 0.001

**Table 2:** Element content in different structures in the cladding with 15% TiC.

Position	Element Mass Percentage (wt%)					
	Mo	Fe	B	C	Cr	Ti
Point 1	50.14	17.18	18.01	11.11	3.37	0.19
Point 2	10.02	3.97	8.44	20.81	2.56	54.20
Point 3	3.42	60.2	6.92	27.45	1.98	0.03

**Table 3:** Main chemical reactions in weld pool.

Number	Chemical Reaction	Number	Chemical Reaction
1	[Mo] + [B] = MoB	9	7[Cr] + 3[C] = Cr_7_C_3_
2	[Mo] + 2[B] = MoB_2_	10	[Ti] + [C] = TiC
3	2[Mo] + [B] = Mo_2_B	11	[Ti] + [Fe] = FeTi
4	[Fe] + [B] = FeB	12	[Ti] + 2[Fe] = Fe_2_Ti
5	2[Fe] + [B] = Fe_2_B	13	[Fe] + 2MoB = Mo_2_FeB_2_
6	[Fe] + FeB = Fe_2_B	14	2[Mo] + 2FeB = Mo_2_FeB_2_ + [Fe]
7	[Cr] + [B] = CrB	15	2[Mo] + 2Fe_2_B = Mo_2_FeB_2_ + 3[Fe]
8	[Cr] + 2[B] = CrB_2_		
